# Modeling solutions for microbial water contamination in the global south for public health protection

**DOI:** 10.3389/fmicb.2025.1504829

**Published:** 2025-04-02

**Authors:** Sylvester Chibueze Izah, Matthew Chidozie Ogwu

**Affiliations:** ^1^Department of Community Medicine, Faculty of Clinical Sciences, Bayelsa Medical University, Yenagoa, Nigeria; ^2^Goodnight Family Department of Sustainable Development, Living Learning Center, Appalachian State University, Boone, NC, United States

**Keywords:** microbial contamination, predictive modeling, public health, environmental health, waterborne diseases, water safety, sustainable water management

## Abstract

Microbial contamination of water sources is a pressing global challenge, disproportionately affecting developing regions with inadequate infrastructure and limited access to safe drinking water. In the Global South, waterborne pathogens such as bacteria, viruses, protozoa, and helminths contribute to diseases like cholera, dysentery, and typhoid fever, resulting in severe public health burdens. Predictive modeling emerges as a pivotal tool in addressing these challenges, offering data-driven insights to anticipate contamination events and optimize mitigation strategies. This review highlights the application of predictive modeling techniques—including machine learning, hydrological simulations, and quantitative microbial risk assessment —to identify contamination hotspots, forecast pathogen dynamics, and inform water resource allocation in the Global South. Predictive models enable targeted actions to improve water safety and lower the prevalence of waterborne diseases by combining environmental, socioeconomic, and climatic factors. Water resources in the Global South are increasingly vulnerability to microbial contamination, and the challenge is exacerbated by rapid urbanization, climate variability, and insufficient sanitation infrastructure. This review underscores the importance of region-specific modeling approaches. Case studies from sub-Saharan Africa and South Asia demonstrated the efficacy of predictive modeling tools in guiding public health actions connected to environmental matrices, from prioritizing water treatment efforts to implementing early-warning systems during extreme weather events. Furthermore, the review explores integrating advanced technologies, such as remote sensing and artificial intelligence, into predictive frameworks, highlighting their potential to improve accuracy and scalability in resource-constrained settings. Increased funding for data collecting, predictive modeling tools, and cross-sectoral cooperation between local communities, non-governmental organizations, and governments are all recommended in the review. Such efforts are critical for developing resilient water systems capable of withstanding environmental stressors and ensuring sustainable access to safe drinking water. By leveraging predictive modeling as a core component of water management strategies, stakeholders can address microbial contamination challenges effectively, safeguard public health, and contribute to achieving the United Nations’ Sustainable Development Goals.

## 1 Introduction

Microbial contamination of water sources is a pressing global issue that poses significant challenges to public health, particularly in the Global South, because their unique socioeconomic and environmental challenges amplify its impact (Erinle et al., 2021; Izah et al., 2023, 2022a,b). High levels of fecal contamination in drinking water, particularly in rural areas of Africa and Southeast Asia, are a significant concern (Ovuru et al., 2023; Sawyer et al., 2023). Inadequate water, sanitation, and hygiene conditions contribute significantly to waterborne diseases, with contaminated water being a key vector. WHO estimates that 5% of all deaths in developing countries stem from water-related diseases, emphasizing the need for effective water quality monitoring and interventions (Prüss-Ustün et al., 2014). The World Health Organization (WHO) estimates that over 2 billion people lack access to safe drinking water, leading to many health risks associated with waterborne pathogens (Ramírez-Castillo et al., 2015). WHO also estimates that 5% of all deaths in developing countries stem from water-related diseases, emphasizing the need for effective water quality monitoring and interventions (Prüss-Ustün et al., 2014).

In parts of the Global South, contaminated water is a major contributor to diseases such as cholera, dysentery, and typhoid fever, which disproportionately affect vulnerable populations in low-income regions (Ramírez-Castillo et al., 2015). The challenges of ensuring safe water supplies in developing countries are exacerbated by inadequate infrastructure, rapid urbanization, and climate change, leading to increased flooding and contamination of water sources (Hering et al., 2013). In many cases, the existing water treatment facilities are outdated or insufficient to handle the growing demand and the complexity of contaminants present in the water supply (Hering et al., 2013). The public health risks associated with microbial contamination are profound (Ben-Eledo et al., 2017; Enaregha et al., 2022; Agedah et al., 2015; Seiyaboh et al., 2020a,b), as contaminated water can lead to severe morbidity and mortality, particularly among children and immunocompromised individuals (Ramírez-Castillo et al., 2015). The disease burden attributable to waterborne pathogens is immense, with millions of cases reported annually, highlighting the urgent need for effective interventions (Ramírez-Castillo et al., 2015). Additionally, the economic implications of waterborne diseases are significant, as they can lead to increased healthcare costs and lost productivity, further straining the limited resources of developing nations (Mekonnen and Hoekstra, 2016).

Many communities in the Global South lack access to improved water sources, and the assumption that these sources present no risk is often misguided (Bain et al., 2014a,b). For instance, studies have shown that even improved water sources can harbor pathogens, particularly in regions with inadequate infrastructure (Poulin et al., 2020; Iyiola et al., 2023a, 2024a,b). This highlights the necessity for comprehensive water safety plans incorporating local knowledge and practices to effectively manage water quality and mitigate risks associated with microbial contamination (Leftwich et al., 2021). Traditional culture-based water testing methods are often impractical in the Global South due to resource constraints and delayed results, underscoring the value of rapid methods like multiplex real-time polymerase chain reaction for timely and accurate pathogen detection (Gemeda et al., 2022; Ogwu and Kosoe, 2024). Socio-economic disparities exacerbate the issue, as many communities lack access to safe water sources, and even “improved” sources may harbor pathogens due to poor infrastructure (Bain et al., 2014b). Comprehensive water safety plans incorporating local knowledge and practices are crucial for managing risks effectively (Leftwich et al., 2021). Environmental factors, including seasonal variations, further complicate microbial contamination dynamics, as seen in studies from Uganda (Sadik et al., 2017; Erhunmwunse et al., 2024). Predictive models must integrate these variables to guide public health strategies and resource allocation. Addressing microbial water contamination in the Global South requires advanced detection technologies, community-driven approaches, and robust public health policies tailored to local contexts to ensure sustainable and impactful solutions.

Predictive modeling emerges as a vital tool in managing water quality, particularly in anticipating contamination events and optimizing mitigation strategies. Predictive modeling involves using mathematical and computational techniques to simulate the behavior of water systems under various conditions, allowing for the identification of potential contamination sources and assessing their impacts (Perelman et al., 2012). This approach is particularly relevant in water quality management, enabling stakeholders to make informed decisions regarding resource allocation and intervention strategies (Perelman et al., 2012). By leveraging predictive modeling, water management authorities can anticipate contamination events, implement timely responses, and allocate resources more effectively, enhancing public health protection (Perelman et al., 2012). The public health risks associated with microbial contamination in water sources are profound (Izah and Ineyougha, 2015; Izah et al., 2021a), as contaminated water can lead to severe morbidity and mortality, particularly among vulnerable populations such as children and immunocompromised individuals (Odiyo et al., 2020). The burden of disease attributable to waterborne pathogens is immense, with millions of cases reported annually, underscoring the urgent need for effective interventions (Prüss-Ustün et al., 2014). Unsafe drinking water is a significant contributor to diarrhea and is responsible for an estimated 10% of global mortality among children under five (Prüss-Ustün et al., 2014). Moreover, the economic implications of waterborne diseases are significant, as they can lead to increased healthcare costs and lost productivity, further straining the limited resources of developing nations (Peletz et al., 2016). The economic burden is compounded by many low-income countries lacking adequate water quality monitoring systems, which can exacerbate the public health crisis (Peletz et al., 2016).

Predictive modeling emerges as a vital tool in managing water quality, particularly in anticipating contamination events and optimizing mitigation strategies. This approach employs mathematical and computational techniques to simulate the behavior of water systems under various conditions, allowing for the identification of potential contamination sources and the assessment of their impacts (Scanlon M. et al., 2022; Bentham and Whiley, 2018). For example, quantitative microbial risk assessment (QMRA) has effectively evaluated the risks associated with microbial contamination in drinking water, providing valuable insights into potential health impacts (Bentham and Whiley, 2018; Abuzerr, 2024). By leveraging predictive modeling, water management authorities can anticipate contamination events, implement timely responses, and allocate resources more effectively, enhancing public health protection (Scanlon B. R. et al., 2022; Bentham and Whiley, 2018). This proactive approach is particularly relevant in regions where waterborne diseases are prevalent, as it enables stakeholders to make informed decisions regarding resource allocation and intervention strategies (Ahmed et al., 2020). Furthermore, the integration of predictive modeling with real-time data collection can significantly improve the responsiveness of water quality management systems. For instance, the use of rapid indicators for microbial contamination can facilitate quicker assessments of water safety, allowing for immediate public health interventions (Colford et al., 2012). This is crucial in areas prone to extreme weather events, exacerbating water quality issues and increasing the risk of waterborne disease outbreaks (Cann et al., 2012). The combination of predictive modeling and real-time monitoring enhances the capacity to manage water quality and fosters a more resilient public health infrastructure capable of addressing the challenges posed by microbial contamination (Cann et al., 2012; Bentham and Whiley, 2018).

This paper aims to explore the application of predictive modeling as a tool to mitigate microbial contamination in water sources, with a specific focus on solutions tailored for developing countries facing resource constraints. Given the unique challenges faced by these regions, it is imperative to develop cost-effective and sustainable modeling approaches that can inform water management practices and enhance the resilience of water supply systems (Mekonnen and Hoekstra, 2016). The scope of this review involves the evaluation of existing modeling frameworks, identifying gaps in current practices, and formulating recommendations for implementing predictive modeling in the context of microbial water contamination.

## 2 Microbial contaminants in water sources in the global south

Microbial contaminants in water sources represent a global public health concern, particularly in developing countries where sanitation and hygiene practices are often inadequate. A complex interplay of environmental, socio-economic, and health factors shapes microbial contaminants in Global South water sources. This region faces significant water quality challenges, directly impacting public health. These contaminants include a variety of microorganisms, such as bacteria, viruses, protozoa, and other pathogens, that can lead to serious health issues when ingested through contaminated water (Table 1). The primary types of microbial contaminants found in water sources include pathogenic bacteria like *Escherichia coli*, viruses such as norovirus, and protozoa like *Giardia* and *Cryptosporidium* (Murphy et al., 2015; Gwimbi et al., 2019). Each of these pathogens has distinct sources and modes of transmission, contributing to the overall burden of waterborne diseases. One of the primary concerns is the prevalence of fecal contamination in drinking water sources. Studies have shown that even improved water sources, such as protected wells and piped supplies, often harbor significant microbial pathogens. Many supposedly improved sources are frequently contaminated, particularly in rural areas of Africa and Southeast Asia, where the risk of waterborne diseases is disproportionately high (Bain et al., 2014a,b). This situation is exacerbated by inadequate sanitation infrastructure and poor hygiene practices, prevalent in many communities across the Global South (Bain et al., 2014b). The health implications of microbial contamination are severe, particularly for vulnerable populations such as children. Waterborne diseases, including cholera, dysentery, and typhoid fever, are major contributors to morbidity and mortality in these regions. For example, Luby et al. (2015) found a direct correlation between microbial contamination in drinking water and the incidence of diarrhea among children in Bangladesh, emphasizing the urgent need for adequate water quality management. Furthermore, as reported by Kumar et al. (2012), the presence of antibiotic-resistant bacteria in water sources raises additional concerns about the effectiveness of treatment options for infections stemming from contaminated water.

**TABLE 1:** Microbial contaminants of water sources in the global south that are of global public health concern.

Microbial contaminant	Type	Source of contamination	Associated diseases	Affected regions	Public health impact	References
*Escherichia coli*	Bacteria	Fecal contamination from humans and animals	Diarrhea, dysentery, urinary tract infections	Sub-Saharan Africa, South Asia	It is a leading cause of bacterial diarrhea, especially in children under 5 years old.	Gwimbi et al., 2019
*Vibrio cholerae*	Bacteria	Contaminated water, poor sanitation	Cholera	East Africa, South Asia, Latin America	It causes cholera outbreaks, leading to dehydration and death if untreated. Affects millions annually.	Jutla et al., 2015
*Salmonella typhi*	Bacteria	Sewage-contaminated water and food	Typhoid fever	South Asia, Sub-Saharan Africa	This leads to high morbidity and mortality if untreated; and commonly causes enteric fever in developing regions.	Crump et al., 2004
*Shigella* spp.	Bacteria	Fecal-oral transmission through contaminated water	Shigellosis (bacillary dysentery)	Sub-Saharan Africa, South Asia	A major cause of dysentery, particularly among children, contributes significantly to child mortality.	Kotloff et al., 2013
Hepatitis A Virus	Virus	Ingestion of contaminated water or food	Hepatitis A	Global, particularly in low-income regions	Causes liver inflammation; outbreaks occur in areas with poor sanitation and hygiene practices.	Jacobsen and Wiersma, 2010
Norovirus	Virus	Contaminated food and water, person-to-person contact	Gastroenteritis	Worldwide	A common cause of viral gastroenteritis outbreaks; it is highly contagious and spreads rapidly in communities.	Patel et al., 2008
*Giardia lamblia*	Protozoa	Contaminated water, especially untreated surface water	Giardiasis	Sub-Saharan Africa, Latin America, South Asia	It causes gastrointestinal symptoms and malabsorption, prevalent in areas lacking clean drinking water.	Thompson and Monis, 2012
*Cryptosporidium* spp.	Protozoa	Contaminated water sources (surface and recreational)	Cryptosporidiosis	Global, particularly in Sub-Saharan Africa	Causes severe diarrhea, particularly in immunocompromised individuals such as those with HIV/AIDS.	Kotloff et al., 2013
*Entameba histolytica*	Protozoa	Fecally contaminated water and food	Amebiasis	South Asia, Sub-Saharan Africa, Latin America	It causes dysentery and liver abscesses; it is endemic in areas with poor sanitation.	Stanley, 2003
*Rotavirus*	Virus	Contaminated water and surfaces, person-to-person contact	Severe diarrhea, vomiting, dehydration	Global, especially in developing regions	A leading cause of severe diarrhea in children contributes to high child mortality rates in low-income countries.	Estes and Kapikian, 2007
*Campylobacter* spp.	Bacteria	Animal feces contaminating water sources	Campylobacteriosis	Worldwide, especially in low-income regions	Causes gastrointestinal illness; a leading cause of bacterial gastroenteritis globally.	Blaser et al., 2008
*Leptospira* spp.	Bacteria	Contaminated water, especially from animal urine	Leptospirosis	Southeast Asia, Latin America, Africa	It causes fever, jaundice, kidney damage, and meningitis and spreads in flood-prone areas.	Adler and de la Peña Moctezuma, 2010
*Schistosoma* spp.	Helminth	Contaminated freshwater sources (snail vectors)	Schistosomiasis	Sub-Saharan Africa, Middle East, Southeast Asia	It causes chronic infection, leading to liver and bladder damage; it affects millions annually in water-contact activities.	Colley et al., 2014
*Ascaris lumbricoides*	Helminth	Contaminated soil and water	Ascariasis	Sub-Saharan Africa, Latin America, Southeast Asia	Intestinal parasitic infection affects nutritional status, especially in children; it is prevalent in areas with poor sanitation.	Bethony et al., 2006
*Dracunculus medinensis*	Helminth	Contaminated drinking water from copepods (water fleas)	Guinea worm disease	West Africa, parts of Asia	Near eradication, but it still poses a threat in some communities; it causes severe pain and long-term disability.	Hopkins et al., 2008

Although the sources of these microbial contaminants in Global South water are multifaceted, the key contributors include sewage discharge, agricultural runoff, and poor sanitation practices. Sewage often introduces a variety of pathogens into water bodies, particularly in areas lacking adequate wastewater treatment facilities (Sibiya and Gumbo, 2013; T’Seole et al., 2022). Agricultural runoff, which may contain fertilizers and animal waste, can also contaminate surface water sources, exacerbating the risk of waterborne diseases (Murphy et al., 2015; Gwimbi et al., 2019). Furthermore, inadequate sanitation infrastructure, particularly in rural and impoverished urban areas, creates conditions conducive to spreading pathogens through water sources (T’Seole et al., 2022; Medeiros and Ferreira, 2020). The interrelationship between these sources and the resultant microbial contamination underscores the need for comprehensive water management strategies addressing water quality and sanitation.

The health impacts of microbial contamination in water are profound, particularly in developing countries with limited access to clean water. Waterborne diseases such as cholera, typhoid fever, and dysentery are prevalent in these regions, leading to significant morbidity and mortality (Jutla et al., 2015; Prüss-Ustün et al., 2014). For instance, cholera outbreaks have been linked to contaminated drinking water sources, with studies indicating that improved sanitation and water purification can drastically reduce the incidence of such diseases (Jutla et al., 2015; Armah et al., 2018). The World Health Organization estimates that inadequate water, sanitation, and hygiene (WASH) contribute to approximately 10% of the global disease burden, with diarrheal diseases alone accounting for a substantial portion of this statistic (McGinnis et al., 2019). In terms of mortality, it is estimated that around 2.2 million people die each year from diarrheal diseases linked to unsafe drinking water and poor sanitation (Prüss-Ustün et al., 2014). Statistics on morbidity and mortality associated with contaminated water highlight the urgent need for improved water quality management. For example, in sub-Saharan Africa, it is estimated that over 300,000 children under five die each year from diarrheal diseases linked to unsafe water and inadequate sanitation (Armah et al., 2018). Moreover, the economic burden of waterborne diseases is significant, with healthcare costs and lost productivity due to illness placing a strain on already limited resources in developing nations (Minh and Hùng, 2011; Patel et al., 2013). The interplay between health impacts and economic factors emphasizes the necessity for investment in water and sanitation infrastructure to improve public health outcomes.

In addition to health risks, the variability of microbial contamination due to environmental factors poses further challenges. Seasonal changes can significantly influence pathogen concentrations in water sources, as demonstrated by Sadik et al. (2017), who noted fluctuations in pathogen levels in Kampala, Uganda, during different seasons. This variability necessitates robust monitoring and modeling efforts to effectively predict contamination events and inform public health responses (Sokolova et al., 2012; Wu H. et al., 2021). Moreover, the Global South’s socio-economic context complicates the water quality issue. Many communities rely on untreated surface water or poorly maintained water supply systems, susceptible to contamination from agricultural runoff, industrial discharges, and inadequate waste management practices (Ahmed et al., 2020). The reliance on boiling water as a treatment method, as observed in Sikkim, India, indicates a common practice among communities to mitigate health risks. However, eliminating all pathogens may not always be sufficient (Singh et al., 2019). It is essential to incorporate sophisticated monitoring methods, including microbial source tracking and hydrodynamic modeling, to comprehend and treat the origins of contamination (Sokolova et al., 2012; Wu J. et al., 2021). These methods can improve water safety and public health outcomes in the Global South by assisting in identifying sources of contamination and providing guidance for focused remedies.

Despite recognizing these issues in the Global South, current challenges in controlling waterborne pathogens remain significant. Inadequate water infrastructure and sanitation facilities are prevalent in many regions, particularly in low-income countries where funding for such projects is often insufficient (T’Seole et al., 2022; Minh and Hùng, 2011). This lack of infrastructure not only hampers access to clean water but also increases the risk of contamination from various sources, including untreated sewage and agricultural runoff (Sibiya and Gumbo, 2013; T’Seole et al., 2022). Furthermore, limited access to real-time water quality monitoring and testing exacerbates the problem, as communities may remain unaware of harmful pathogens in their water supply (Nefale et al., 2017). The absence of effective surveillance systems makes it challenging to implement timely interventions to mitigate the risks associated with microbial contamination. Improving water quality and sanitation must also consider the socio-economic factors influencing access to these essential services. Communities with low socio-economic status often face more significant challenges in securing safe drinking water and adequate sanitation facilities, affecting their overall health and well-being. The relationship between poverty, water insecurity, and health outcomes is well-documented, indicating that addressing these disparities is crucial for reducing the burden of waterborne diseases. Additionally, community participation in sanitation initiatives has been shown to enhance the effectiveness of interventions, as local involvement can lead to more sustainable practices and better maintenance of facilities (Olugbamila et al., 2020; O’Reilly, 2015). Some of the world’s most polluted rivers include the Amazon, Ganges, Yangtze, and Niger Rivers, highlighting the shared challenges they face due to industrial, agricultural, and domestic pollution (Fearnside, 2016; Central Pollution Control Board (CPCB)., 2019; Ma et al., 2022). The Amazon River is significantly affected by deforestation and mining, while the Ganges suffers from untreated sewage and industrial discharge, prompting initiatives like the “Namami Gange” program aimed at rejuvenation (Ministry of Water Resources., 2019). The Yangtze River, impacted by industrial waste and ecological changes from the Three Gorges Dam, has seen the implementation of stringent regulations to protect its waters (Ma et al., 2022). Meanwhile, the Niger River faces pollution from oil spills and agricultural runoff, leading to regional cooperation efforts for sustainable practices (Ifelebuegu et al., 2017). This underscores the importance of cross-regional learning, suggesting that successful strategies from one river can inform management practices in others, thereby enhancing the overall understanding of global water pollution and its mitigation.

## 3 Overview of predictive modeling in water quality management

### 3.1 Concepts of predictive modeling

Predictive modeling in environmental science is a critical tool for understanding and managing microbial water contaminants. These models are designed to forecast the presence and concentration of microbial pathogens in water systems, thereby assisting in public health protection and environmental management (Table 2). The primary purpose of predictive models is to provide insights into the dynamics of microbial contamination, enabling stakeholders to make informed decisions regarding water quality management and risk assessment. By utilizing various data inputs, including environmental variables, land use patterns, and historical contamination data, predictive models can simulate potential contamination scenarios and assess the effectiveness of intervention strategies (Saeidi et al., 2018a; Wu J. et al., 2021).

**TABLE 2:** Predictive modeling in environmental science for managing microbial water contaminants.

Modeling technique	Application	Advantages	Limitations	Example tools	References
Machine learning (ML)	Predicting contamination events and sources in water systems	High accuracy with large datasets; adaptive to new data	Requires large, high-quality datasets for training	TensorFlow, Scikit-learn, and Keras	Scanlon M. et al., 2022
Quantitative microbial risk assessment (QMRA)	Assessing microbial contamination risks in water sources	Quantifies health risks inform policy and interventions	Complex modeling requires extensive environmental and health data	@Risk, QMRAcatch, and RStudio	Ahmed et al., 2020
Hydrological modeling	Simulating water flow and transport of microbial contaminants	Helpful in understanding ecological processes	Requires significant computational resources and hydrological data	SWAT (Soil and Water Assessment Tool), and MIKE-SHE	Wu H. et al., 2021
Geographic information systems (GIS)	Mapping contamination sources and high-risk areas	Spatial analysis capability: helpful in visualizing data	Requires integration with other data sources for complete accuracy	ArcGIS and QGIS	Schijven et al., 2013a,b
Regression analysis	Predicting relationships between microbial indicators and environmental variables	Simple to implement and interpret	Assumes linearity; may not capture complex relationships	SPSS, R (glm function), and MATLAB	Saeidi et al., 2018b
Stochastic models	Accounting for variability and uncertainty in microbial contamination	Captures randomness and uncertainty in environmental systems	Can be computationally expensive; requires accurate parameter estimation	@Risk, and Crystal Ball	Cann et al., 2012
Agent-based modeling (ABM)	Simulating the behavior of individual entities (e.g., pathogens, human populations) in water systems	Provides a detailed understanding of complex interactions	Requires detailed data and computational resources	NetLogo, and AnyLogic	Bentham and Whiley, 2018
Time series analysis	Predicting future contamination events based on historical data	Effective for forecasting based on past trends	It does not capture sudden, unprecedented changes in contamination	R (forecast package), Python (statsmodels), and MATLAB	Perelman et al., 2012
Bayesian networks	Probabilistic modeling of contamination risks	Incorporates uncertainty and prior knowledge	Requires detailed knowledge and probabilistic data	GeNIe, Netica, and BayesiaLab	Tian et al., 2020
Artificial neural networks (ANN)	Learning complex patterns in contamination events	Capable of modeling non-linear relationships	Requires large datasets and computational power	TensorFlow, PyTorch, and Keras	Kuroki et al., 2023

Several types of predictive models are employed in the context of microbial water contaminants, each with distinct methodologies and applications. Statistical models, such as regression analysis, are commonly used to identify relationships between microbial indicators and environmental factors. For instance, it demonstrated that incorporating land use categories and chemical tracers significantly improved model performance for predicting fecal indicators in urban environments, achieving R^2^ values greater than 0.69 (Saeidi et al., 2018b). These statistical approaches allow for quantifying the impact of various environmental factors on microbial water quality, providing a basis for targeted interventions. Machine learning techniques have also gained prominence in predictive modeling for microbial contamination. Wu J. et al. (2021) utilized machine learning algorithms to track significant sources of water contamination, highlighting the importance of hydrologic features and land cover in predicting microbial sources. This approach allows for the integration of large datasets and complex interactions between variables, enhancing the accuracy of predictions. Furthermore, machine learning models can adapt to new data, improving their predictive capabilities and providing real-time insights into water quality dynamics.

Hydrological models represent another category of predictive tools for assessing microbial contamination in water bodies. These models simulate the movement of water through the environment, accounting for factors such as precipitation, runoff, and groundwater flow. ’s study on groundwater contamination risks from pit latrines illustrates how hydrological models can be employed to predict microbial contamination based on various environmental parameters, including soil type and groundwater table variations (Hinton, 2023). Stakeholders can better manage water resources and mitigate contamination risks by understanding the hydrological processes that contribute to microbial transport. Developing all-encompassing strategies to combat microbiological water contamination requires integrating different modeling approaches. For instance, combining statistical models with machine learning techniques can enhance predictive accuracy by leveraging the strengths of both methodologies. Additionally, incorporating hydrological modeling into predictive frameworks allows for a more holistic understanding of how microbial contaminants move through ecosystems, ultimately informing management practices to reduce public health risks. The significance of predictive modeling extends beyond academic research; it has practical implications for water quality management and public health protection. For example, predictive models can inform the design of monitoring programs by identifying critical times and locations for sampling, thereby optimizing resource allocation. Moreover, these models can assist in evaluating the effectiveness of interventions, such as implementing best management practices in agricultural settings or establishing buffer zones around water bodies (Holvoet et al., 2012).

Furthermore, predictive modeling is crucial in risk assessment related to microbial water contamination. These models can guide regulatory decisions and public health interventions by estimating the likelihood of contamination events and their potential health impacts. For instance, Atta et al. (2019) highlighted the relationship between drinking water contamination and gastrointestinal illnesses, underscoring the need for practical predictive tools to prevent outbreaks. By quantifying the risks associated with different contamination scenarios, stakeholders can prioritize actions to safeguard public health. In addition to traditional modeling approaches, technological advancements have facilitated the development of innovative predictive tools. For example, remote sensing and geographic information systems (GIS) have enhanced the ability to monitor environmental determinants of microbial contamination in recreational waters. Kotchi et al. (2015) emphasized the importance of integrating Earth observation systems to detect microbial contamination promptly, which is critical for protecting public health during recreational activities. These technological advancements enable more efficient data collection and analysis, ultimately improving the reliability of predictive models. Moreover, the incorporation of climate data into predictive models has become increasingly relevant in the context of microbial water quality. Xu et al. (2019) demonstrated that climate and land use factors significantly influence bacterial levels in stormwater, highlighting the need for models that account for changing environmental conditions. As climate change continues to impact water quality, predictive modeling will be essential for understanding and mitigating the effects of these changes on microbial contamination.

**FIGURE 1:**
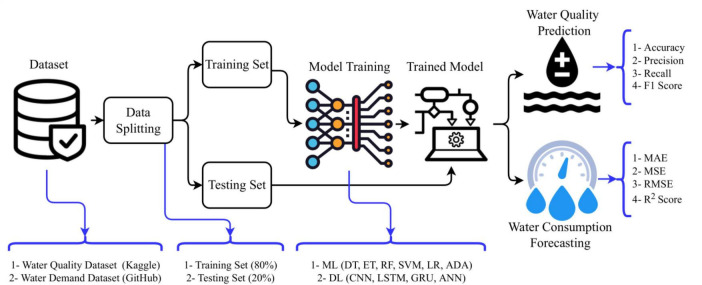
Framework for predicting water quality and consumption using machine learning and deep learning models. Source: Rustam et al. (2022).

**FIGURE 2:**
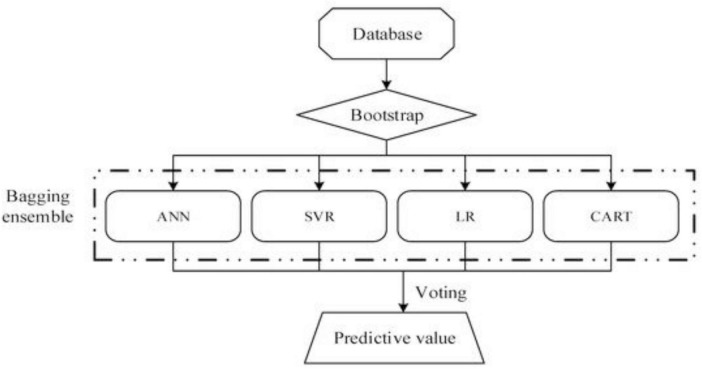
Bagging ensemble model for enhanced predictive analytics of microbes in water. Source: Chou et al. (2019).

Figure 1 illustrates a conceptual framework for predicting water quality and consumption, leveraging machine learning (ML) and deep learning (DL) models by Rustam et al. (2022). The process typically begins with dataset collection from two sources: a water quality dataset (Kaggle) and a water demand dataset (GitHub). These datasets are split into training (80%) and testing (20%) subsets to build and validate predictive models. The ML techniques employed include Decision Trees (DT), Extra Trees (ET), Random Forests (RF), Support Vector Machines (SVM), Logistic Regression (LR), and Adaptive Boosting (ADA). On the other hand, the DL methods include Convolutional Neural Networks (CNN), Long Short-Term Memory (LSTM), Gated Recurrent Units (GRU), and Artificial Neural Networks (ANN). The trained models are evaluated for water quality prediction using metrics like Accuracy, Precision, Recall, and F1 Score. Simultaneously, water consumption forecasting is assessed using Mean Absolute Error (MAE), Mean Squared Error (MSE), Root Mean Squared Error (RMSE), and R^2^ Score. This approach effectively monitors water resources, providing actionable insights into quality and consumption patterns. Using advanced algorithms enables the framework to enhance prediction accuracy, facilitating data-driven decision-making for sustainable water resource management. Another relevant framework is a bagging ensemble framework integrating multiple machine learning models for predictive analytics (Figure 2; Chou et al., 2019). This model is essential and suitable for the Global South because it can handle missing data. The process begins with a database where data is bootstrapped to create diverse training datasets. These datasets are fed into different predictive models, including Artificial Neural Networks (ANN), Support Vector Regression (SVR), Linear Regression (LR), and Classification and Regression Trees (CART). Combining these models, the bagging ensemble leverages their strengths and reduces prediction variance. The final predictive value is obtained through a voting mechanism aggregating all models’ outputs, ensuring a robust and accurate prediction. From an application standpoint, this approach is highly beneficial in scenarios requiring high precision, such as water quality monitoring, environmental risk assessments, or industrial process optimization. The ensemble model’s capability to handle diverse data types and mitigate overfitting enhances its adaptability to real-world problems, particularly in dynamic systems with complex variables.

Diverse LSTM models are widely used for microbial prediction because they process sequential data and capture temporal dependencies. Standard LSTMs predict microbial growth based on time-series inputs like pH, temperature, and nutrient levels, while Bidirectional LSTMs (BiLSTMs) enhance accuracy by analyzing data in forward and backward directions. Stacked LSTMs, with multiple layers, learn complex patterns for dynamic environments or multi-stage processes. Models with attention mechanisms prioritize critical input features, improving predictions under varied conditions. Convolutional LSTMs (ConvLSTMs) handle spatial-temporal data, such as microbial biofilm growth, and hybrid LSTMs combine techniques like SVMs for enhanced accuracy. These models support critical applications in food safety, industrial optimization, and environmental health management. Also, the innovative use of the Hyperconic Multilayer Perceptron (HC-MLP) for predicting microbial growth under varying conditions, such as pH levels and nutrient concentrations, has been experimented with by Murrieta-Dueñas et al. (2021). By leveraging experimental data from *Pseudomonas aeruginosa*, the HC-MLP offers a novel approach to microbial growth modeling. Its ability to create complex non-linear decision boundaries enhances the accuracy of predictions compared to traditional models. The method supports reduced experimental costs, optimizes the design of bioreactors, and advances real-time control strategies for biological processes, making it particularly valuable for applications in biotechnology, food safety, and pharmaceutical industries? The application of predictive modeling in microbial water quality assessment is not without challenges. One significant issue is the variability in microbial indicators and their relationship with environmental factors. For instance, the presence of fecal coliforms as an indicator of microbial contamination may not always correlate with pathogenic microorganisms, leading to potential misinterpretations of water quality (Sarker et al., 2016). Therefore, ongoing research is necessary to refine predictive models and improve their accuracy in assessing microbial risks.

### 3.2 Steps in developing predictive models

**FIGURE 3:**
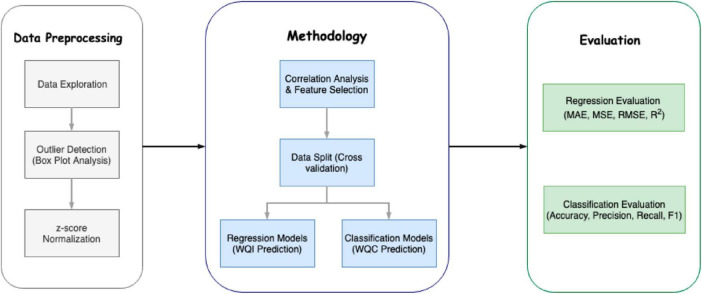
Standard framework for water quality index prediction- data preprocessing, methodology, and evaluation. Source: Ahmed et al. (2019).

Developing predictive models for water contamination involves a systematic approach encompassing several critical steps, including data collection and analysis, model design, calibration, and validation (Figure 3). The framework provides a systematic approach to predicting water quality by integrating robust data preprocessing, advanced modeling techniques, and comprehensive evaluation metrics. By incorporating outlier detection and z-score normalization, the framework ensures data integrity and readiness for analysis. Regression models for Water Quality Index prediction and classification models for water quality classification allow for tailored analysis of continuous and categorical outcomes (Table 3). Note that water quality classification thresholds often vary based on regional or institutional standards, though the provided ranges are widely recognized in environmental assessments. However, this classification system is a valuable tool for policymakers and environmental managers to identify water quality issues and prioritize appropriate remedial actions. Additionally, adapting these thresholds to reflect specific socio-economic and ecological contexts is essential for addressing unique regional challenges effectively. The Water Quality Index integrates multiple parameters to evaluate water quality comprehensively. Physical parameters, such as temperature, turbidity, and total dissolved solids, assess water clarity and usability. Chemical parameters, including pH, dissolved oxygen, biological oxygen demand, and contaminants like nitrates and phosphates, measure chemical impacts on ecosystems and health. Microbiological parameters, such as fecal and total coliform, indicate microbial contamination and disease risks. Heavy metals like lead, arsenic, and mercury assess toxicity, while nutrient levels (ammonia, nitrites, phosphorus) highlight risks of eutrophication. Toxic substances, including pesticides and PCBs, trace industrial and agricultural pollutants, while indicators like hardness, alkalinity, and chlorides provide general water quality insights for various uses. Incorporating metrics such as MAE, RMSE, Accuracy, and F1 Score ensures the reliability and validity of the predictions. This structured methodology is particularly valuable in addressing the complexities of water quality assessment, offering insights that can inform public health interventions and resource management. This process is essential for ensuring that the models accurately reflect the complexities of hydrological systems and can effectively predict water quality outcomes.

**TABLE 3:** Water quality classification categories based on Water Quality Index values.

QI Range	Classification	Water quality description	Suitability for use
0–25	Excellent	Very high-quality water with minimal contamination.	Suitable for drinking, irrigation, and aquatic life without treatment.
26–50	Good	Clean water with minor contamination levels.	Suitable for most uses, including drinking after rudimentary treatment.
51–75	Fair	Moderately polluted water; potential risks to health if untreated.	Suitable for irrigation and industrial use, it requires significant treatment for drinking.
76–100	Poor	Highly polluted water with considerable contamination.	It is unsuitable for drinking and may support limited industrial or agricultural use.
>100	Very Poor/unsuitable	Severely polluted water is hazardous to health and ecosystems.	It is not suitable for any purpose and requires extensive treatment before use.

### 3.3 Data collection and analysis

The first step in developing predictive models is comprehensive data collection, which involves gathering various environmental, meteorological, and hydrological data types. This data is the foundation for model development and can include temperature, precipitation, land use, and water quality indicators. For instance, the Soil Water Assessment Tool (SWAT) utilizes extensive datasets related to weather, soil properties, topography, and vegetation to simulate water quantity and quality in complex watersheds (Bacu et al., 2011; Iyiola et al., 2023b). Similarly, machine learning models for predicting *Escherichia coli* loads integrate hydrometeorological data alongside animal density and grazing patterns, demonstrating the importance of diverse datasets in enhancing predictive accuracy (Abimbola et al., 2020). In addition to raw data, the analysis phase is crucial for understanding the relationships between different variables and identifying patterns that can inform model development. Statistical methods and exploratory data analysis techniques are often employed to discern correlations and trends within the data. For example, regression methods have been utilized to predict *E. coli* levels based on various environmental factors, highlighting the role of statistical analysis in shaping predictive models (Abimbola et al., 2020). Furthermore, the integration of machine learning techniques allows for the identification of complex non-linear relationships that traditional statistical methods may overlook, thus improving the robustness of predictions (Ahmed et al., 2019).

### 3.4 Model design, calibration, and validation

Once the data has been collected and analyzed, the next step involves designing the predictive model. This phase includes selecting the appropriate modeling framework and algorithms that align with the study’s specific objectives. For instance, hydrodynamic and water quality models such as EPANET and SWAT are commonly used in water contamination studies due to their ability to effectively simulate hydraulic and water quality processes (Cervantes, 2023; Bacu et al., 2011). EPANET has been enhanced with various extensions to improve its capabilities in modeling water quality under different conditions, including pressure-dependent demand scenarios (Seyoum et al., 2013). Calibration is a critical step in model development, wherein the model parameters are adjusted to ensure that the model outputs align with observed data. This process often involves iterative testing and refinement, utilizing historical data to fine-tune the model’s predictive capabilities. For example, the calibration of the SWAT model has been extensively documented, with studies demonstrating its effectiveness in simulating nutrient runoff and sediment yields in various watersheds (Ali et al., 2020). Similarly, the calibration of EPANET models is essential for accurately predicting water quality outcomes, particularly in large distribution networks where computational efficiency is paramount (Davis et al., 2018). Validation follows calibration and assesses the model’s predictive performance against independent datasets. This step is crucial for establishing the model’s credibility and ensuring it can reliably predict future water quality scenarios. Various validation techniques, including cross-validation and hold-out testing, evaluate model accuracy and generalizability (Towett et al., 2020). For instance, the validation of machine learning models for predicting water quality has highlighted the importance of using diverse datasets to ensure that the models perform well across different environmental conditions (Ahmed et al., 2019).

### 3.5 Key predictive models used in water contamination studies

Various predictive models are employed in water contamination studies, each with unique strengths and applications (Table 4). These models provide essential tools for advancing water quality prediction and decision-making in environmental management. Hydrodynamic and water quality models, such as SWAT and EPANET, are widely recognized for their ability to simulate the transport and fate of contaminants in water systems. SWAT, for example, is particularly effective in assessing the impact of land management practices on water quality, making it a valuable tool for watershed management (Bacu et al., 2011; Femeena et al., 2020). EPANET, on the other hand, excels in modeling water distribution systems and has been enhanced with various extensions to improve its functionality in simulating water quality dynamics (Cervantes, 2023; Seyoum et al., 2013). In addition to traditional hydrodynamic models, statistical and machine learning approaches have gained prominence in recent years for predicting pathogen presence and water quality. Machine learning algorithms, such as support vector machines and random forests, have been successfully applied to predict *E. coli* presence in tap water, demonstrating their effectiveness in handling complex datasets and identifying key predictors of water quality (Kuroki et al., 2023). These models leverage large volumes of data to uncover patterns that may not be immediately apparent through conventional statistical methods, thus providing a more nuanced understanding of water contamination dynamics (Banerjee et al., 2022). Moreover, the integration of machine learning with traditional modeling approaches has led to the development of hybrid models that capitalize on the strengths of both methodologies. For instance, combining hydrodynamic models with machine learning techniques allows for improved water quality predictions under varying environmental conditions, as demonstrated in studies that assess the impact of climate change on water quality (Park et al., 2013). This hybrid approach enhances predictive accuracy and provides valuable insights into the interactions between different environmental factors and water quality outcomes.

**TABLE 4:** Some predictive models useful in water contamination studies.

Model type	Model name	Application in water contamination	Key strengths
Machine learning	Decision tree	Classifying contamination sources and predicting water quality indices.	Easy interpretability, low computational cost.
	Random forest (RF)	Detecting contamination hotspots and estimating pollutant levels.	High accuracy, handles non-linearity well.
	Support vector machine	Predicting water quality and pathogen presence.	Effective for high-dimensional data.
Deep learning	Convolutional neural network	Analyzing spatial water quality data and remote sensing imagery.	Excellent for image and spatial data.
	Long short-term memory (LSTM)	Modeling temporal patterns of contamination.	Captures time dependencies in datasets.
Regression models	Multiple linear regression	Estimating relationships between pollutants and water quality metrics.	Simple and interpretable.
	Logistic regression	Predicting the probability of contamination events.	Works well for binary classification.
Hybrid models	Ensemble models	Combining ML algorithms for better predictions (e.g., RF + LSTM).	Improves accuracy and reduces bias/variance.
Bayesian models	Bayesian networks	Risk assessment and probabilistic modeling of contamination scenarios.	Accounts for uncertainty and dependencies.

## 4 Application of predictive modeling to mitigate microbial contamination in developing countries

Predictive modeling helps forecast microbial contamination in water systems, offering a valuable tool for improving water quality management in developing countries. By providing data-driven insights, these models assist policymakers and public health officials make informed decisions to mitigate contamination risks and safeguard public health. This section focuses on applying predictive modeling to mitigate microbial contamination in developing countries (Table 5).

**TABLE 5:** Application of predictive modeling to mitigate microbial contamination in developing countries.

Country/region	Predictive modeling application	Outcomes	Challenges	References
Bangladesh	Machine learning models for predicting microbial contamination in groundwater	15% reduction in waterborne diseases in affected rural communities	Lack of quality data for remote areas; infrastructure limitations	Scanlon M. et al., 2022
Nigeria	Quantitative microbial risk assessment for contamination risk in drinking water	Improved identification of high-risk areas, allowing better intervention planning	Poor monitoring infrastructure; lack of funding for wide-scale application	Izah et al., 2021a,b
Kenya	GIS-based risk mapping for microbial contamination in surface water sources	Enabled targeted interventions, reducing contamination in high-risk areas	Limited access to geospatial data and analysis tools	Schijven et al., 2013a,b
South Africa	Hydrological modeling to track microbial contaminants in river systems	Enhanced understanding of contamination pathways, improving mitigation strategies	High computational costs; reliance on accurate hydrological data	Wu H. et al., 2021
India	Bayesian network modeling for microbial risk prediction in drinking water distribution systems	Helped prioritize interventions and infrastructure upgrades to reduce microbial contamination	The complexity of integrating varied data sources (e.g., health, environmental)	Tian et al., 2020
Peru	Agent-based modeling for simulating human-pathogen interactions in rural water systems	Improved understanding of human behaviors influencing contamination risk, leading to educational campaigns	Lack of detailed behavioral and environmental data	Bentham and Whiley, 2018
Vietnam	Time series analysis for predicting microbial contamination in urban wastewater systems	Enabled proactive management of wastewater treatment systems, preventing contamination peaks	Difficulties in long-term monitoring; high variability in water quality	Perelman et al., 2012
Ethiopia	Artificial neural networks for microbial contamination prediction in surface water sources	Increased predictive accuracy in identifying contamination hotspots	Computational limitations in resource-poor settings	Kuroki et al., 2023
Ghana	Regression analysis of microbial contamination in urban watersheds	Identification of land-use patterns contributing to microbial contamination	Assumes linear relationships that may not always exist	Saeidi et al., 2018a
Brazil	Stochastic modeling for understanding variability in microbial contamination levels	Improved risk assessment and management of microbial contamination in urban slums	Lack of accurate parameter estimates and computational resources	Cann et al., 2012

### 4.1 Identifying risk zones for microbial contamination in global south water sources

Identifying risk zones for microbial contamination is critical to public health, particularly in developing countries where infrastructure may be inadequate to manage water safety effectively. Predictive models have emerged as a powerful tool in mapping high-risk contamination areas, particularly those near sewage systems and flood-prone regions. Such predictive models leverage historical data and environmental factors to identify areas at heightened risk for microbial contamination, thereby allowing for implementing proactive measures. For instance, studies have shown that regions near sewage discharge points are significantly more susceptible to contamination from fecal indicator bacteria, which can lead to gastrointestinal diseases (Soller et al., 2014; Schoen and Ashbolt, 2010). Furthermore, flood-prone regions often experience runoff that can introduce pathogens into water supplies, necessitating the integration of predictive modeling to assess risk (Viau et al., 2011).

One of the primary challenges in identifying risk zones is the significant variability in microbial contamination across different geographical areas. Studies have highlighted that fecal contamination is more prevalent in rural areas than urban settings, particularly in regions of Africa and Southeast Asia, where many communities rely on unimproved water sources (Bain et al., 2014a,b). This disparity underscores the necessity for targeted interventions in rural zones, where the risk of waterborne diseases is heightened due to inadequate sanitation infrastructure and limited access to safe drinking water (Bain et al., 2014b). Environmental factors also play a crucial role in microbial contamination. For instance, rainfall has been shown to correlate with increased bacterial contamination in groundwater, as microorganisms can be mobilized from contaminated surfaces into water supplies during heavy rains (Bagordo, 2024). This relationship suggests that seasonal variations must be considered when mapping risk zones, as periods of heavy rainfall may exacerbate contamination levels and increase the risk of waterborne disease outbreaks (Bagordo, 2024). Additionally, anthropogenic activities, such as industrial discharges and agricultural runoff, contribute to the microbial load in water bodies, as demonstrated by Posada-Perlaza et al. (2019) who found that human contamination in the Bogotá River significantly altered microbial communities and promoted the spread of antibiotic resistance genes. Moreover, socio-economic factors significantly influence the risk of microbial contamination. Communities with limited resources often lack the infrastructure for effective waste management and water treatment, leading to increased exposure to pathogens (Bain et al., 2014a,b). For example, the reliance on untreated surface water sources in many rural areas heightens the risk of contamination from nearby agricultural or industrial activities. This situation necessitates a multi-faceted approach to risk zone identification that incorporates socio-economic data alongside environmental assessments to effectively target interventions (Bain et al., 2014a,b). Furthermore, advancements in technology and methodologies can enhance the identification of risk zones. Geographic Information Systems (GIS) and remote sensing technologies offer powerful tools for mapping microbial contamination and identifying at-risk areas based on environmental determinants (Kotchi et al., 2015). These technologies can facilitate the integration of various data sources, including hydrological models and microbial community assessments, to provide a comprehensive understanding of contamination dynamics (Kotchi et al., 2015). In conclusion, the Global South’s perspective on identifying risk zones for microbial contamination in water sources emphasizes the need for a holistic approach that integrates environmental, socio-economic, and technological factors. Addressing these challenges is essential for developing effective public health strategies that can mitigate the risks associated with microbial contamination and improve water safety in vulnerable communities.

Integrating spatial data through GIS with microbial risk assessments enhances the ability to visualize and analyze contamination risks effectively. GIS allows for the layering of various data types, such as population density, land use, and historical contamination events, to create comprehensive risk maps (Schijven et al., 2013a,b; Sulistyawati, 2020). This spatial analysis can identify current risk zones and potential future risks based on environmental changes and urban development (Ogwu, 2019). For example, GIS has successfully been employed in dengue control programs to monitor mosquito populations and predict outbreaks, demonstrating its utility in managing infectious diseases (Schijven et al., 2013a,b). Similarly, applying GIS in water safety assessments can provide valuable insights into the dynamics of microbial contamination, enabling targeted interventions in high-risk areas (Sulistyawati, 2020).

### 4.2 Predicting microbial water contamination events and outbreaks in the global south

Predicting contamination events and outbreaks is another vital area where predictive modeling plays a significant role. Real-time prediction of microbial contamination following extreme weather events, such as floods and droughts, is crucial for timely public health responses. Extreme weather can disrupt sanitation systems and increase runoff, carrying pathogens into water supplies (Awwad et al., 2019). Predictive models incorporating meteorological data can forecast potential contamination events, allowing for preemptive measures, such as issuing boil water advisories or deploying rapid response teams to affected areas (Chang et al., 2021). Early-warning systems based on these predictive models can significantly reduce the incidence of waterborne diseases by facilitating rapid public health interventions (Viau et al., 2011).

Another significant aspect of predicting contamination events is the role of environmental conditions, particularly rainfall and land use. Studies have shown that heavy rain can lead to increased runoff, which often carries pathogens from agricultural and urban areas into water sources (Wu H. et al., 2021; Cann et al., 2012). For instance, Wu J. et al. (2021) utilized machine learning techniques to track significant sources of water contamination, highlighting how hydrological features and land cover influence microbial sources. This approach underscores the necessity of considering local environmental dynamics when predicting contamination risks. Moreover, socio-economic factors play a crucial role in the vulnerability of communities to waterborne diseases. Many areas in the Global South lack adequate sanitation infrastructure, which exacerbates the risk of contamination during extreme weather events (Cann et al., 2012). For example, the reliance on surface water sources, which are more susceptible to contamination, significantly increases the likelihood of outbreaks, particularly in low-income populations (Ahmed et al., 2020). This relationship between socioeconomic conditions and water quality highlights the need for targeted interventions addressing environmental and community-level vulnerabilities. Technological advancements also offer promising avenues for improving predictions of microbial contamination. Applying QMRA can help estimate the likelihood of waterborne disease outbreaks based on various risk factors, including water quality and exposure levels (Ahmed et al., 2020). Additionally, remote sensing and GIS integration can facilitate real-time monitoring and modeling of microbial contamination events, enabling timely public health responses (Wu J. et al., 2021; Weiskerger et al., 2019).

### 4.3 Resource allocation and decision support for effective management of microbial contaminants in global south water

Resource allocation and decision support are essential to effective public health management, particularly in resource-limited settings. Predictive models can prioritize interventions by identifying at-risk populations and areas and optimizing resource allocation. For instance, quantitative microbial risk assessments (QMRA) can be utilized to estimate the health impacts of various intervention strategies, allowing decision-makers to allocate resources more effectively (Cohen et al., 2021; Ahmed et al., 2010). Decision-making tools incorporating predictive modeling can guide community water treatment and sanitation improvements, ensuring that interventions are practical and efficient (Cohen et al., 2021). By leveraging data-driven insights, public health officials can make informed decisions that enhance water safety and reduce the burden of microbial diseases.

Effective resource allocation is essential for addressing the widespread issue of microbial contamination in the Global South. Bain et al. (2014b) highlight that many water sources, including piped systems and wells, are often contaminated, necessitating targeted interventions to improve water quality. The variability in contamination levels across different source types underscores the importance of context-specific strategies prioritizing resources for the most affected communities (Kumpel et al., 2016). For instance, areas with high levels of fecal contamination require immediate attention to mitigate health risks associated with waterborne diseases (Bain et al., 2014a,b). Decision support systems that leverage data and technology can enhance the management of microbial contaminants. Using QMRA provides a framework for understanding the relationship between water quality and health outcomes, enabling stakeholders to make informed decisions regarding resource allocation (Clasen et al., 2015). Additionally, advancements in microbial source tracking can help identify contamination sources, allowing for targeted interventions that address specific risks (Esselman et al., 2018). These tools can inform public health policies and guide investments in water infrastructure and sanitation improvements (Clasen et al., 2015; Wolf et al., 2018). Community engagement is also vital for effective resource allocation and decision-making. Involving local populations in assessing water quality and identifying contamination sources fosters a sense of ownership and accountability, which can enhance the sustainability of interventions (Kumpel et al., 2016). Moreover, education and awareness campaigns can empower communities to adopt better hygiene practices and advocate for improved water management (Prüss-Ustün et al., 2014).

### 4.4 Case studies from developing countries

The application of predictive modeling to mitigate microbial contamination in developing countries has gained significant traction, particularly in the context of water safety. This is especially pertinent in regions such as sub-Saharan Africa and parts of Asia, where waterborne diseases like cholera pose substantial public health risks. The success stories of predictive modeling applications in these areas illustrate the potential of technology to enhance water safety and public health outcomes. Similar predictive modeling efforts have been employed in sub-Saharan Africa to address water safety concerns. For example, in sub-Saharan Africa, predictive models have been used to prevent cholera outbreaks by identifying high-risk areas based on environmental and demographic data (Viau et al., 2011; Cohen et al., 2021). These models have enabled health authorities to implement targeted vaccination campaigns and improve sanitation infrastructure in vulnerable communities. By analyzing land use patterns and hydrological features, researchers could predict the presence of fecal contaminants in drinking water sources. This information was crucial for local health officials, who could prioritize water quality testing and remediation efforts in areas identified as high-risk (Wu H. et al., 2021; Ijabadeniyi et al., 2011). Such applications not only enhance the safety of drinking water but also empower communities to take charge of their water quality management. Controlling water pollution and implementing effective remediation strategies are critical for safeguarding aquatic ecosystems and public health. A multifaceted approach is essential, beginning with the establishment of stringent water quality standards and the implementation of monitoring systems to ensure compliance with these standards (Ntsasa et al., 2024). Best management practices (BMPs) in agriculture, such as the creation of riparian buffer strips and the adoption of sustainable land-use practices, can significantly reduce non-point source pollution, which is a major contributor to water quality degradation (Malaj et al., 2014; Tong et al., 2011). Moreover, the integration of advanced technologies, such as IoT-based water quality monitoring systems, can facilitate real-time tracking of pollution levels and enhance response strategies (Pasika and Gandla, 2020). In urban areas, improved stormwater management practices, including green infrastructure and treatment systems, can mitigate runoff and its associated pollutants (Nwokediegwu et al., 2024). Additionally, the use of innovative remediation techniques, such as nanoremediation, has shown promise in effectively removing contaminants from water bodies (El-Ramady et al., 2017). Collaborative efforts among stakeholders, including governments, industries, and local communities, are vital for developing comprehensive water management plans that address both pollution prevention and remediation (Posthuma et al., 2019; Rout et al., 2022). Employing a combination of regulatory measures, technological advancements, and community engagement, it is possible to create sustainable solutions for controlling water pollution and restoring affected ecosystems (Rout et al., 2022). In parts of Asia, predictive modeling has been used to assess the risk of waterborne diseases following monsoon seasons, allowing for timely interventions that significantly reduce disease incidence (Viau et al., 2011; Cohen et al., 2021). By integrating data on rainfall, temperature, and historical cholera cases, models can predict the likelihood of an outbreak occurring in specific regions. This proactive approach has enabled health authorities to allocate resources more effectively and implement timely interventions, such as vaccination campaigns and public health messaging, reducing cholera’s incidence significantly (Wu J. et al., 2021; Ding et al., 2017). The lessons learned from these implementations highlight the challenges and successes associated with predictive modeling in developing countries. One significant challenge is the availability and quality of data. In many regions, especially rural areas, data on water quality, environmental conditions, and health outcomes may be sparse or unreliable. This lack of data can hinder the development of robust predictive models. However, innovative approaches, such as remote sensing and citizen science, have emerged to address these data gaps. For example, mobile applications that allow community members to report water quality issues can provide valuable real-time data for predictive modeling efforts (Árvai and Post, 2011). Another challenge is the integration of predictive models into existing public health frameworks. Successful implementation requires collaboration among various stakeholders, including government agencies, non-governmental organizations, and local communities. Effective communication and training ensure health officials can interpret and apply model outputs in decision-making processes. In some cases, pilot projects have demonstrated the effectiveness of predictive modeling, leading to broader adoption and integration into public health strategies (Árvai and Post, 2011; Ding et al., 2017).

Moreover, the sustainability of predictive modeling initiatives is a critical consideration. Many projects face funding constraints and may rely on external support. To address this, some researchers advocate for developing local capacity through training programs that empower communities to independently maintain and operate predictive modeling systems. This approach enhances local ownership and ensures that predictive modeling efforts can continue to evolve and adapt to changing environmental and health conditions (Árvai and Post, 2011). In addition to these challenges, the successes of predictive modeling applications in developing countries underscore the importance of interdisciplinary collaboration. Integrating environmental science, public health, and data science expertise is crucial for developing effective predictive models. Collaborative efforts can lead to more comprehensive approaches considering the multifaceted nature of water safety and public health (Wu H. et al., 2021; Wu J. et al., 2021; Árvai and Post, 2011). Furthermore, the role of technology in enhancing predictive modeling capabilities cannot be overstated. Advances in artificial intelligence and machine learning have significantly improved the accuracy and efficiency of predictive models. For instance, using neural networks and time series analysis has enabled researchers to develop models that can predict water quality changes with greater precision, considering various environmental factors (Wu et al., 2023). These technological advancements hold promise for further enhancing the effectiveness of predictive modeling in addressing microbial contamination in water supplies.

### 4.5 Modeling antimicrobial resistance (AMR) genes in water

Modeling AMR genes in water is a critical and growing aspect of health surveillance, particularly given the increasing prevalence of antimicrobial-resistant bacteria (ARB) in aquatic environments. The presence of antimicrobial resistance genes (ARGs) in water bodies poses significant public health risks, as these genes can be transferred among bacteria, leading to the emergence of multi-drug resistant pathogens. This phenomenon is exacerbated by anthropogenic activities, including the discharge of untreated or inadequately treated wastewater into rivers and lakes, which serve as reservoirs for these resistance genes (Guerra et al., 2022; Cutrupi et al., 2024; Waseem et al., 2017). Recent studies have highlighted the importance of monitoring ARGs in various water sources, including urban drinking water and sewage systems. For instance, Guerra et al. (2022) demonstrated the occurrence of both antimicrobial residues and ARGs in urban drinking water and sewage in Southern Brazil, emphasizing the need for improved sewage treatment and monitoring systems. Similarly, Bueno et al. (2021) employed spatial mapping to quantify and predict the presence of antimicrobials and ARGs in Minnesota’s water bodies, underscoring the role of environmental factors in the spread of AMR. These findings indicate that water bodies can act as significant reservoirs for ARGs, facilitating their dissemination into broader ecosystems (Ogura et al., 2020).

Metagenomic approaches have emerged as powerful tools for advancing the understanding of AMR in aquatic environments. Ottesen et al. (2022) utilized metagenomic and quasimetagenomic methods to analyze surface waters, revealing a complex resistome that is influenced by local anthropogenic activities, such as proximity to hospitals. This aligns with the findings of Hendriksen et al. (2019) who conducted metagenomic analyses of urban sewage, highlighting the critical role of wastewater in the global spread of AMR. Such methodologies not only provide insights into the diversity of ARGs present but also help in assessing the potential health risks associated with exposure to contaminated water sources. The environmental persistence of ARGs is further complicated by the selective pressure exerted by the presence of antimicrobials in water. Studies have shown that exposure to disinfectants like chlorine can enhance the expression of resistance genes in microbial populations, thereby increasing the survival and proliferation of ARB in treated water (Karumathil et al., 2014; Hayward et al., 2020). This phenomenon is particularly concerning in wastewater treatment plants (WWTPs), which are often hotspots for horizontal gene transfer among bacteria (Kotlarska et al., 2014). The implications of these findings are profound, as they suggest that even treated water can harbor significant levels of AMR, posing risks to human health and the environment.

## 5 Data and technological challenges in developing countries

Developing countries face significant challenges in data collection and technological infrastructure, which hinder effective environmental management and decision-making. Limited access to reliable data, inadequate technological tools, and lack of skilled personnel contribute to difficulties in addressing ecological issues. Some the key data and technological challenges in developing countries are outlined in Table 6.

**TABLE 6:** Key data and technological challenges in developing countries.

Challenge	Examples	Impact on predictive modeling	References
Data availability	Lack of reliable and up-to-date water quality monitoring data	Reduces the accuracy of models and limits the ability to predict contamination events	Wu H. et al., 2021
Data quality	Inconsistent or inaccurate environmental and health data	This leads to unreliable model outputs and ineffective interventions	Schijven et al., 2013a
Limited computational resources	Lack of access to high-performance computing infrastructure	Inhibits the use of complex models such as machine learning and neural networks	Kuroki et al., 2023
Technological infrastructure	Limited access to essential technologies such as sensors, GIS tools, and computational software	Restricts the real-time monitoring and updating of models	Ahmed et al., 2020
Skilled personnel	Shortage of trained professionals with expertise in data science and predictive modeling	Slows down the adoption and implementation of advanced modeling techniques	Cann et al., 2012
Funding and investment	Insufficient funding for large-scale data collection and technological investments	Limits the ability to scale up predictive modeling efforts	Izah et al., 2021b
Integration of multiple data sources	Difficulty in integrating diverse datasets such as land use, hydrological, and health data	Reduces model efficiency and ability to make holistic predictions	Tian et al., 2020
Sensor and data collection limitations	Lack of adequate water quality sensors for real-time data collection	Prevents timely detection of contamination and undermines proactive intervention	Bentham and Whiley, 2018
Internet connectivity and digital divide	Poor internet infrastructure in rural areas impedes cloud-based modeling and real-time collaboration	Hinders the ability to implement advanced, centralized modeling systems	Perelman et al., 2012
Policy and governance gaps	Lack of clear policies around water quality data collection and management	This leads to inconsistent practices and delayed responses to contamination risks	Saeidi et al., 2018a

### 5.1 Data gaps and availability

In low-resource settings, the issues surrounding incomplete or unreliable water quality data are profound and multifaceted. The lack of comprehensive data hampers effective decision-making and policy formulation, leading to adverse public health outcomes. For instance, highlight that incomplete water event data significantly undermines the management of transboundary river basins, which is critical for ensuring water quality and availability in China and neighboring countries (Wang and Lv, 2022). Similarly, the study emphasizes that chronic environmental contamination, such as that from per- and poly-fluoroalkyl substances, exacerbates community stress and complicates public health responses due to inadequate data on contamination levels (Calloway et al., 2020). This lack of reliable data affects immediate public health interventions and undermines long-term strategies for sustainable water resource management. To address these data gaps, innovative approaches to data collection are essential. Mobile-based reporting systems have emerged as a viable solution, enabling real-time data collection and reporting from communities directly affected by water quality issues. Such systems can enhance the accuracy and timeliness of data, as demonstrated in various pilot projects across developing regions. Community involvement is another critical component; engaging local populations in monitoring efforts can foster a sense of ownership and responsibility toward water resources. Phungela et al. (2022) argued that effective water quality monitoring is vital for identifying contributors to water quality variations, which can inform integrated water resource management strategies. By leveraging local knowledge and participation, data collection efforts can become more robust and reflective of actual conditions on the ground.

Moreover, integrating community-driven data collection with mobile technology can enhance the reliability of water quality assessments. This approach democratizes data collection and empowers communities to actively safeguard their water resources. The use of participatory methods in data collection has been shown to yield more accurate and contextually relevant information, thereby improving the overall quality of data available for public health decision-making (Zhang and Wang, 2022). As such, addressing data gaps through innovative and community-focused strategies is crucial for mitigating the impacts of microbial contamination in developing countries.

### 5.2 Technological limitations

Technological limitations pose significant challenges to the practical application of predictive modeling in resource-constrained environments. Limited access to advanced technologies and computational infrastructure restricts the ability of public health officials and researchers to develop and implement sophisticated models that can predict water quality and contamination levels. For instance, the reliance on outdated technologies can lead to inefficiencies in data processing and analysis, as highlighted by the findings of Helly et al. (2021), who noted that even in relatively developed regions, uncertainties in data quality can hinder effective decision-making. This situation is exacerbated in low-resource settings where access to modern computational tools is often severely limited. To overcome these technological barriers, strategies such as cloud computing and the use of open-source platforms can be employed. Cloud computing offers a scalable solution that allows for the storage and processing of large datasets without the need for significant local infrastructure investment. This approach can facilitate collaborative research efforts and enable access to advanced analytical tools that would otherwise be unavailable in resource-limited settings. Furthermore, open-source platforms can democratize access to modeling tools, allowing local researchers and public health officials to adapt and utilize these resources according to their specific needs. Zhang and Wang (2022) emphasize the importance of building social resilience in public health governance, which includes leveraging technology to enhance data collection and analysis capabilities. Additionally, adapting existing modeling tools to fit the constraints of low-resource environments is crucial. This may involve simplifying models to reduce computational demands while providing valuable insights into water quality dynamics. Integrating local knowledge and expertise into these models can also enhance their relevance and applicability. By fostering an environment where local researchers can contribute to model development, the predictive capabilities of these tools can be significantly improved, leading to better-informed public health interventions (Bergeron et al., 2017). Ultimately, addressing technological limitations through innovative strategies is essential for enhancing the effectiveness of predictive modeling in mitigating microbial contamination in developing countries.

### 5.3 Training and capacity building

Building local expertise in predictive modeling and data interpretation is paramount for addressing the challenges of microbial contamination in water resources. The importance of training and capacity-building initiatives cannot be overstated, as they empower local public health officials, water authorities, and community members to utilize data and modeling tools effectively. As highlighted by Galway et al. (2016), interdisciplinary research capacity is essential for addressing complex public health challenges, including those related to water quality. Without adequate training, local stakeholders may struggle to interpret data accurately or apply predictive models effectively, leading to suboptimal decision-making. Capacity-building programs should be tailored to the needs of public health officials and water authorities in developing countries. These programs can include workshops, online courses, and hands-on training sessions focusing on predictive modeling techniques, data analysis, and interpretation. For instance, Meredith (2023) emphasized the need for facilitated online learning to build strategic public health skills, which can be particularly beneficial in resource-constrained environments. By enhancing the skills of local practitioners, these initiatives can foster a more informed and capable workforce better equipped to tackle water quality issues.

Moreover, community involvement in capacity-building efforts is crucial for ensuring the sustainability of these initiatives. Engaging local communities in training programs can enhance their understanding of water quality issues and empower them to monitor and manage their water resources actively. This participatory approach builds local capacity and fosters a sense of ownership and responsibility toward water quality management. The findings highlight the importance of building capability within the community and public health workforce to ensure sustainable public health interventions (Adams and Dickinson, 2010).

## 6 Public health implications of predictive modeling

### 6.1 Enhancing disease surveillance and control

Integrating predictive models with public health surveillance systems is paramount for enhancing disease surveillance and control, particularly in waterborne diseases. Predictive modeling leverages historical data and real-time information to forecast disease outbreaks, enabling public health officials to implement timely interventions. For instance, the application of artificial intelligence (AI) in public health surveillance systems has shown significant promise in identifying and containing infectious diseases, particularly waterborne pathogens (Adefemi, 2023). By utilizing predictive analytics, health authorities can anticipate outbreaks based on environmental factors, historical trends, and population movements, optimizing resource allocation and response strategies. Moreover, integrating predictive models into public health frameworks can guide waterborne disease outbreak response strategies. The systematic review by Wang and Yang (2019) highlighted the effectiveness of remote sensing techniques in monitoring water quality, which is crucial for predicting potential outbreaks of waterborne diseases. Such monitoring systems can provide comprehensive data on water quality indicators, allowing for proactive management measures. This proactive approach is essential in developing countries where inadequate surveillance systems and limited resources often exacerbate the burden of waterborne diseases. By combining predictive modeling with public health surveillance, authorities can enhance their capacity to respond to outbreaks, ultimately reducing morbidity and mortality associated with waterborne diseases. The role of community engagement in improving disease surveillance cannot be overstated. Cohen et al. (2019) emphasized the importance of social capital in a community’s recovery during emergencies, suggesting that community engagement skills among emergency responders can significantly improve public health outcomes. By fostering trust and communication within communities, public health officials can enhance the effectiveness of surveillance systems, ensuring that individuals are more likely to report illnesses and adhere to public health recommendations. This community-centered approach is particularly vital in developing countries, where cultural factors and trust in health systems can significantly influence disease reporting and response efforts.

### 6.2 Supporting sustainable water management

Predictive modeling plays a critical role in long-term water quality monitoring and sustainable management, particularly in the context of achieving the Sustainable Development Goals (SDGs). SDG 6 emphasizes the importance of ensuring the availability and sustainable management of water and sanitation for all. The Integrated Monitoring Initiative for SDG 6 (IMI-SDG 6) provides a comprehensive framework for monitoring water quality indicators, including ambient water quality, which is essential for assessing progress toward this goal (Wu, 2023). By employing predictive models, stakeholders can identify trends in water quality over time, enabling them to implement targeted interventions to mitigate pollution and improve water management practices. Additionally, predictive tools can facilitate the achievement of water-related SDGs by providing insights into the interlinkages between water quality and other sustainable development objectives. For example, the research by Alcamo (2019) highlighted the synergies between water quality and various SDGs, suggesting that improvements in water quality can positively impact health, education, and economic development. By integrating predictive modeling into water management strategies, policymakers can better understand these interlinkages and develop comprehensive approaches that address multiple SDGs simultaneously. The importance of effective water governance is also underscored in the context of sustainable water management. Improved water governance supports social, economic, and environmental objectives, as highlighted by (Bertule et al., 2018). By utilizing predictive models to assess governance progress, stakeholders can identify areas for improvement and implement strategies that enhance the resilience of water systems. This is particularly relevant in developing countries, where governance challenges often hinder effective water management and contribute to water quality issues.

### 6.3 Promoting community health and resilience

Using predictive models to empower communities with information on water safety and contamination risks is essential for promoting community health and resilience. By providing communities with real-time data on water quality, stakeholders can enhance public awareness and encourage proactive measures to mitigate contamination risks. For instance, Imani et al. (2021) demonstrated the feasibility of integrating historical data and machine learning techniques to develop resilience predictive models for water quality. Such models can inform communities about potential risks, enabling them to take preventive actions and improve their resilience to waterborne diseases. Building resilient water systems is crucial for protecting vulnerable populations in developing countries. The research by Allen et al. (2021) emphasized the importance of integrating public health considerations into water infrastructure planning, particularly in climate change and its impacts on water resources. By adopting a holistic approach that considers the interconnections between water infrastructure, public health, and community resilience, stakeholders can develop better systems to withstand environmental stresses and respond to public health emergencies. Furthermore, community resilience is closely linked to the availability and accessibility of healthcare services. Cohen et al. (2020) highlighted the role of healthcare facilities in building community resilience, particularly during emergencies. When communities have confidence in the availability of health services, they are more likely to engage in preventive health behaviors and report health issues, ultimately enhancing community resilience. This underscores the need for integrated approaches that combine water management, public health, and community engagement to foster resilience in vulnerable populations.

## 7 Policy and institutional frameworks for predictive modeling in water management

Integrating predictive modeling in water management is increasingly recognized as critical for effective governance and sustainable resource management. This integration is heavily influenced by policy frameworks that provide the necessary support and guidance for adopting and implementing predictive modeling techniques. Such frameworks are essential for establishing standards, facilitating data sharing, and promoting collaboration among various stakeholders involved in water management. The importance of these policy frameworks cannot be overstated, as they align the interests of different organizations, ensuring that predictive modeling is adopted and effectively utilized to address the challenges posed by microbial contaminants in water systems.

Governmental and international organizations play a vital role in fostering an environment conducive to data sharing and collaboration. By establishing policies that encourage transparency and cooperation among stakeholders, these entities can enhance the effectiveness of predictive modeling initiatives. For instance, as Dewulf et al. (2011 discussed), collaborative frameworks emphasize connecting various actors’ perspectives to effectively address complex water governance issues. Establishing such frameworks is crucial in overcoming barriers to collaboration, which can often stem from fragmented governance structures and differing organizational priorities (Widmer et al., 2019). Moreover, international organizations usually provide the technical expertise and financial resources necessary for implementing predictive modeling projects, thereby facilitating the sharing of best practices and lessons learned across borders (Feng, 2023). Institutional collaboration is another critical aspect of effectively implementing predictive modeling in water management. Partnerships between governments, research institutions, and non-governmental organizations (NGOs) can significantly enhance data collection, analysis, and interpretation capacity. These partnerships allow for the pooling of resources and expertise, which is particularly important in addressing the multifaceted challenges of microbial contamination in water systems. For example, the collaborative governance model proposed by Widmer et al. (2019) highlighted the importance of social-ecological fit in managing water quality across different jurisdictions. This model underscores the need for interconnected governance arrangements that can adapt to the complexities of water management, particularly in transboundary contexts where multiple stakeholders are involved.

International agencies also play a vital role in providing technical and financial support for predictive modeling initiatives. These agencies often facilitate capacity-building efforts, enabling local governments and organizations to develop the necessary skills and knowledge to implement predictive modeling effectively. The strategic vision for international collaboration outlined by Feng (2023) emphasized the importance of establishing partnerships and sharing experiences to enhance resource management capabilities. Furthermore, global organizations can assist in developing common standards and guidelines that promote consistency and reliability in predictive modeling practices, fostering stakeholder trust and encouraging broader participation in collaborative efforts (Boer et al., 2016). Integrating predictive modeling within policy frameworks requires a concerted effort to address the various barriers that may hinder collaboration. As highlighted by Turek et al. (2022), understanding the dynamics of stakeholder engagement is crucial for fostering effective partnerships. This understanding can be achieved through comprehensive stakeholder analyses that identify common interests and facilitate aligning goals among different organizations. Additionally, the role of community management in rural water supplies, as discussed by Opare (2011), illustrated the potential for local communities to engage in collaborative water management efforts, provided they receive adequate support and resources from external agencies. This local engagement is essential for ensuring that predictive modeling efforts are grounded in the realities of the communities they aim to serve.

## 8 Future directions and research needs

### 8.1 Innovations in predictive modeling for water contamination

Integrating emerging technologies such as artificial intelligence (AI) and big data analytics into predictive modeling for water contamination presents a transformative opportunity for water management. These technologies enable the processing and analysis of vast datasets, facilitating real-time monitoring and predictive analytics that can significantly enhance decision-making processes in water quality management (Li et al., 2019). For instance, AI algorithms can identify patterns and predict potential contamination events by analyzing historical water quality data alongside environmental variables (Liu and Yang, 2018). The ability to harness big data analytics allows for integrating diverse datasets, including meteorological, hydrological, and anthropogenic factors, which are crucial for understanding complex water systems (Ma et al., 2020a). Moreover, interdisciplinary research combining water science, public health, and data science is essential for developing robust predictive models that address the multifaceted nature of water contamination. The collaboration between these fields can lead to innovative approaches that predict water quality issues and assess their implications for public health (Golembiewski et al., 2018). For example, integrating public health data with water quality models can help identify vulnerable populations and inform targeted interventions (Zhang et al., 2023). This interdisciplinary approach is vital for creating comprehensive solutions addressing water quality challenges’ environmental and health dimensions (Piffer et al., 2021).

### 8.2 Scaling predictive models for broader application

Scaling successful predictive modeling approaches across different regions requires strategic planning and adaptation to local contexts, particularly in the developing world. One effective strategy involves customizing models to account for regional variability in water quality challenges. This necessitates a thorough understanding of local hydrological conditions, pollution sources, and socio-economic factors influencing water quality (Ma et al., 2020b). For instance, models developed in urban settings may not directly apply to rural areas due to differences in land use, population density, and pollution sources (Garaba et al., 2015). Therefore, localized data collection and model calibration are critical for ensuring the accuracy and relevance of predictive models in diverse contexts (Galway et al., 2016). Additionally, addressing regional variability involves engaging local stakeholders in the modeling process. Collaborative efforts with local communities, government agencies, and non-governmental organizations can enhance the applicability of predictive models by incorporating local knowledge and priorities (Pugas, 2023). This participatory approach fosters ownership of the modeling outcomes and ensures that the solutions developed are culturally and contextually appropriate (Su et al., 2022). Furthermore, leveraging technology such as mobile applications for data collection can facilitate community involvement and enhance the richness of the data used in predictive modeling (Aminia et al., 2021).

### 8.3 Sustainability and long-term impacts

Ensuring the sustainability of predictive modeling efforts beyond initial implementation is critical for water quality management. This involves establishing frameworks for continuously monitoring, evaluating, and adapting models to reflect changing environmental conditions and emerging challenges (Islam and Repella, 2015). Long-term sustainability can be achieved by integrating predictive modeling into existing water management policies and practices, ensuring that these tools are not viewed as standalone solutions but as integral components of a broader water governance framework (Fefferman, 2023). Moreover, measuring the long-term impacts of modeling solutions on water quality and public health is essential for assessing the effectiveness of predictive modeling initiatives. Longitudinal studies that track changes in water quality and health outcomes over time can provide valuable insights into the efficacy of interventions guided by predictive models (Kemp and Nurius, 2015). Such studies should establish causal relationships between modeling outputs and real-world outcomes, reinforcing the importance of data-driven decision-making in water management (Richter et al., 2021).

## 9 Conclusion

The role of predictive modeling in mitigating microbial contamination of water sources in developing countries has emerged as a critical component in addressing public health challenges associated with waterborne diseases. The findings from various studies underscore the effectiveness of predictive models in assessing microbial water quality, identifying contamination sources, and informing water management strategies. For instance, it demonstrated that incorporating land use patterns and chemical tracers significantly enhances the predictive performance of models to forecast microbial fecal indicators, thereby facilitating urban development planning that prioritizes human health protection. Similarly, research illustrates how predictive models can perform empirical risk assessments, allowing for targeted interventions before contamination occurs. These insights highlight the need for advanced modeling techniques to safeguard water quality in vulnerable regions. The broader implications of predictive modeling extend beyond immediate microbial contamination concerns, significantly impacting public health and development. By improving water safety, predictive modeling reduces waterborne diseases, disproportionately affecting children and marginalized populations in developing countries. For example, conducted a systematic review that revealed a strong correlation between fecal contamination in drinking water and the prevalence of waterborne diseases, emphasizing the urgent need for effective monitoring and intervention strategies (Bain et al., 2014a,b). Furthermore, the work indicates that using deep tube wells, less susceptible to surface contamination, has led to a notable decline in childhood diarrhea rates in Bangladesh (Escamilla et al., 2011). These findings collectively reinforce the notion that predictive modeling is not merely a technical tool but a vital strategy for enhancing public health outcomes and fostering sustainable development. Considering these findings, there is an urgent call to action for increased investment in predictive modeling technologies and capacity-building initiatives. Collaborative efforts among governments, NGOs, and local communities are essential to protect vulnerable populations from microbial water contamination. As highlighted by Onda et al. (2012), integrating household water treatment strategies can significantly mitigate health risks associated with contaminated water sources. Moreover, establishing water safety plans, as advocated by Leftwich et al. (2021), is crucial for ensuring the continuous functionality and safety of water supply systems. By prioritizing these investments and collaborative approaches, stakeholders can effectively address the pressing challenges of microbial contamination and enhance the resilience of water systems in developing countries.
